# EXO modifies sucrose and trehalose responses and connects the extracellular carbon status to growth

**DOI:** 10.3389/fpls.2013.00219

**Published:** 2013-06-25

**Authors:** Janina Lisso, Florian Schröder, Carsten Müssig

**Affiliations:** Department Lothar Willmitzer, Max Planck Institute of Molecular Plant Physiology, Universität PotsdamPotsdam, Germany

**Keywords:** EXO, growth, sugar response, trehalose, apoplast

## Abstract

Plants have the capacity to adapt growth to changing environmental conditions. This implies the modulation of metabolism according to the availability of carbon (C). Particular interest in the response to the C availability is based on the increasing atmospheric levels of CO_2_. Several regulatory pathways that link the C status to growth have emerged. The extracellular EXO protein is essential for cell expansion and promotes shoot and root growth. Homologous proteins were identified in evolutionarily distant green plants. We show here that the EXO protein connects growth with C responses. The *exo* mutant displayed altered responses to exogenous sucrose supplemented to the growth medium. Impaired growth of the mutant in synthetic medium was associated with the accumulation of starch and anthocyanins, altered expression of sugar-responsive genes, and increased abscisic acid levels. Thus, EXO modulates several responses related to the C availability. Growth retardation on medium supplemented with 2-deoxy-glucose, mannose, and palatinose was similar to the wild type. Trehalose feeding stimulated root growth and shoot biomass production of *exo* plants whereas it inhibited growth of the wild type. The phenotypic features of the *exo* mutant suggest that apoplastic processes coordinate growth and C responses.

## Introduction

A major challenge for plants is the coordination of supply and demand of carbohydrates and other carbon (C) compounds during the diurnal cycle and under changing environmental conditions. Plants developed regulatory pathways that allow matching C consumption and availability. These pathways imply both long-distance and cell type-specific signaling mechanisms (Rolland et al., 2006). Rapid control of C and energy homeostasis may primarily involve posttranslational modifications of proteins and allosteric control of enzymes, whereas transcriptional regulation and adjustment of growth mediate long-term adaptation.

As plants adapted to lower levels of ambient CO_2_ for millions of years, the current increase of atmospheric CO_2_ concentrations will affect crop yields via its effects on photosynthesis, plant metabolism, and water consumption (Leakey et al., 2009). Plant breeders possibly will be able to harness the CO_2_-enriched environment and increase yield of C3 crops, but there are many unanswered questions. Sink capacity (i.e., the transport, consumption, and storage of sugars and nutrients), maintenance of an optimal C/N balance, or photosynthetic capacity (e.g., Rubisco activity) may limit crop yield, but the regulatory processes at the basis of these physiological phenomena are poorly understood. Consequently, precise strategies to improve crops are difficult.

Although the rising atmospheric CO_2_ levels will impose far-ranging ecological and agricultural changes, a critical shortage of C and energy is a common short-term stress in plants. Low light, defoliation by mechanical injury or insect herbivory, or adverse environmental conditions such as flooding stress and hypoxia can result in poor C assimilation and ATP production. Massive changes in enzymes activities, metabolic pathways, and gene expression patterns take place in response to C starvation and energy shortage (Gibon et al., 2009; Bailey-Serres et al., 2012). However, these adaptations primarily reflect stress responses, and the control of growth in dependency of C availability in the absence of stress may involve fine-tuning mechanisms that differ from short-term survival programs.

The analysis of the regulatory pathways that control C partitioning and starch turnover during diurnal cycles may provide more direct insights into the mechanisms linking C availability and growth (Stitt and Zeeman, 2012). For example, both too rapid and too slow mobilization of starch during the night can result in diminished growth rates. Rapid starch degradation causes C starvation at the end of the night, and slow mobilization of starch during the night implies the retention of C reserves that are not used. The circadian clock is one factor that controls transitory starch accumulation, partly via the control of gene expression patterns (Graf et al., 2010; Farré and Weise, 2012; Ruts et al., 2012).

The evolutionary conserved protein kinase Snf1-Related Protein Kinase 1 (SnRK1) is a major regulator of energy homeostasis in plants. It controls numerous genes, metabolic pathways, and ultimately growth and development (Baena-González and Sheen, 2008). SnRK1 activity is controlled by the energy status, C availability, and additional physiological parameters. SnRK1 activity increases under low-energy conditions and promotes catabolic processes. One important regulative factor of SnRK1 activity is the signaling metabolite trehalose-6-phosphate (T6P) (Zhang et al., 2009). The T6P level is positively associated with the sucrose level (Lunn et al., 2006). High sucrose levels cause high T6P levels which inhibit SnRK1 activity. This inhibition may allow C consumption for anabolic processes and promote growth (Schluepmann et al., 2012). Conversely, low T6P levels under starvation conditions allow reprogramming of metabolism by high SnRK1 activity. T6P may also control starch synthesis via sucrose-dependent activation of AGPase (ADP-glucose pyrophosphorylase) (Lunn et al., 2006).

The target of rapamycin (TOR) pathway represents another highly conserved pathway that is critical for metabolic control and growth of cells. When nutrients are plentiful, TOR activates protein synthesis and growth. When nutrients are scarce, the TOR signaling pathway becomes inactive. This allows the induction of catabolic programs and, under severe starvation, autophagy of cell components. Presumably direct interactions between the plant TOR and SnRK1 signaling pathways exist, and the conservation of SnRKs and TOR in all eukaryotes suggests that these pathways represent an important regulatory module connecting nutrient sensing and cell growth (Smeekens et al., 2010; Robaglia et al., 2012).

The known signaling pathways described above are localized in the cytoplasm, are controlled by the nutrient and energy status of the cell, and control growth by modifying cell cycle progression, protein synthesis, and other processes. However, these pathways may need extensive input of various systemic and local signals to properly adjust metabolism to cellular needs and environmental conditions. For example, cell membrane sensors may sense physical cell wall properties, trigger enhanced delivery of cell wall components, and thus maintain cell wall integrity during growth and pathogen defense (Boisson-Dernier et al., 2011; Cheung and Wu, 2011; Levin, 2011; Wolf et al., 2012). Phytohormones interact with sugar responses, sink-source relations, and metabolism (Eveland and Jackson, 2012).

In addition to the regulatory pathways mentioned above, many mutants were identified that show altered responses to sugars (Rolland et al., 2006). Several of these mutants are also impaired in phytohormone biosynthesis or signaling. Examples include abscisic acid-deficient or abscisic acid-insensitive mutants (e.g., *gin6*/*sun6*/*abi4*, Arenas-Huertero et al., 2000; Huijser et al., 2000; *aba2*/*sis4*/*gin1*, Laby et al., 2000; Cheng et al., 2002), mutants with impaired ethylene production or impaired ethylene signaling (e.g., *etr1-1*, Zhou et al., 1998; *sweetie*, Veyres et al., 2008), mutants with altered brassinosteroid responses (e.g., *bls1*, Laxmi et al., 2004), and mutants with altered sensitivity to multiple phytohormones (e.g., *prl1*, Németh et al., 1998).

We identified the *EXO* and *EXO-LIKE* genes as brassinosteroid-responsive genes that control growth under different environmental conditions (Schröder et al., 2009). The EXL1 protein links C and energy metabolism to growth under suboptimal C availability (Schröder et al., 2011). Although *EXL1* is required for adaptation to C- and energy-limiting growth conditions, it is largely irrelevant for growth and development under standard conditions (Schröder et al., 2011). EXL1 reduces BR-dependent growth under low C availability and presumably shuts down C and energy consumption in frame of a starvation response. The EXL2 and EXL4 proteins may have related functions (Schröder et al., 2012). *EXO* is required for growth under standard growth conditions (Schröder et al., 2009). Loss of *EXO* function resulted in reduced leaf size, fresh weight, dry weight, and root length. Epidermis, palisade, and spongy parenchyma cells of the *exo* mutant were smaller in comparison to the wild type.

The physiological and molecular mode of action of EXO and other members of the protein family is unknown. Here, we provide evidence that EXO modifies sugar response of seedling growth, sugar responsive gene expression, and sugar-dependent anthocyanin, starch, and ABA accumulation. The growth defects of the *exo* mutant are partly normalized by exogenous trehalose. We hypothesize that EXO links the extracellular C status with the intracellular C responses.

## Materials and methods

### Plant materials and generation of transgenic plants

The SALK_098602 line (Alonso et al., 2003) carried a T-DNA insertion in the *EXO* coding sequence and was named *exo*. The DNA insertion site, homozygosity of T-DNA insertion, and impaired gene expression were confirmed (Schröder et al., 2009). Accession Col-0 is the wild-type background and was used as control plants.

A 35S::EXO:HA overexpression construct was established using a Gateway-compatible vector. The EXO coding sequence was amplified using the primers forward 5′ CAC CCC TCT TTC ACT ATT ACA CTT TTC CT 3′ and reverse 5′ GAC CAT AGT AGA GCA AGC CGA C 3′. Sequence analysis revealed 100% identity to the *EXO* cDNA sequence. The PCR fragment was cloned into the pENTR/D-TOPO (Invitrogen, Karlsruhe, Germany) vector, and was used to establish the 35S::EXO:HA fusion construct using the pGWB14 (Nakagawa et al., 2007) vector. The construct was transformed into *exo* mutant plants using the floral-dip method. Insertion of the construct and expression of *EXO* in *exo*/35S::EXO-HA plants were confirmed (Schröder et al., 2009).

### Growth conditions

Seeds for growth experiments were derived from plants that were disseminated and grown in parallel in a greenhouse under standard conditions. Plants were grown on half-concentrated Murashige and Skoog (MS) medium supplemented with or without 0.5% sucrose and solidified with 0.4% (w/v) agarose. After 2 days in the dark and at 4°C, plants were transferred into a growth chamber with a long-day light regime (16 h day [140 μmol m^−2^ s^−1^, 22°C]/8 h night [22°C]) and grown in a randomized manner. After 3 days, the germinated plants where transferred on new one-half concentrated Murashige and Skoog medium supplemented with different concentrations of sucrose, other sugars or sugar analogues and solidified with 0.4% (w/v) agarose. The plants where then transferred back into the same growth chamber. Alternatively, plants were established in soil. Seeds were allowed to germinate and to grow for 2 weeks in controlled growth chambers (7 days: 16 h light [140 μmol m^−2^ s^−1^], 20°C, 75% relative humidity; 8 h night, 6°C, 75% relative humidity; thereafter 7 days: 8 h light [140 μmol m^−2^ s^−1^], 20°C, 60% relative humidity; 16 h night, 16°C, 75% relative humidity). Subsequently, plants were transferred to long-day conditions in a controlled growth chamber (16 h light [140 μmol m^−2^ s^−1^], 20°C, 75% relative humidity; 8 h night, 16°C, 60% relative humidity). All genotypes were grown in the same chamber at the same time in a randomized manner.

### Gene expression analysis

After shoot mass quantification, the plant material was immediately frozen in liquid nitrogen and powderized. Analysis of transcript levels was conducted using the same shoot material that was used for other analyses (i.e., quantification of ABA, anthocyanin, and sugar levels, and determination of trehalase activity).

Total RNA was isolated using the Trizol reagent (Invitrogen). One microgram of total RNA was reverse transcribed with the RevertAid H Minus First Strand cDNA Synthesis Kit (Thermo Scientific) to generate first strand cDNA. Real-time RT-PCR was performed with the Maxima SYBR Green qPCR Master Mix (Thermo Scientific). The cycle threshold values of the *eIF*1 α control gene were subtracted from the respective cycle threshold values of the genes of interest. Other control genes gave similar results (Figures A4, A5). The expression value of the genes at 0.5% sucrose was arbitrarily set to 10, and all other values were adjusted accordingly. Higher numbers indicate higher transcript levels. Primer sequences for quantitative RT-PCR are listed in the Table A1. All primer pairs amplified single products, as shown by the melting temperatures of the amplicons.

### Measurement of ABA levels

ABA extraction was done at 4°C with dimmed light. 50 mg powdered shoot tissue were subjected to lyophilisation for 24 h. Tissue was suspended in 0.5 ml extraction buffer (MeOH containing 2.5 mM citric acid monohydrate and 0.5 mM butylated hydroxytoluene). The extract was incubated for at least 20 h in the dark at 4°C under shaking conditions and centrifuged at 1500×g for 15 min at 4°C. The supernatants were recovered and 2 ml 62.5% extraction buffer/28.5% MeOH was added. C18 Sep-Pak cartridges (Waters, Eschborn, Germany) were equilibrated with 2 ml extraction buffer and subsequently with 1 ml 70% extraction buffer/30% MeOH. Supernatants were passed through a C18 Sep-Pak cartridge. 1 ml 70% MeOH was loaded onto the cartridge and flow-throughs were united. Additional elutions were analyzed exemplarily to check for residual ABA on the cartridge. The eluates were dried in a lyophilizer. Dried samples were resuspended in 1 ml 50 mM Tris buffered saline pH 7.5 (TBS)/MeOH (10:1). Dilutions in TBS of each sample extract or ABA standard (Duchefa) were subjected to analysis by an enzyme immunoassay using the PGR1 kit (Sigma-Aldrich) according to the manufacturer's instruction. The ABA standard was assumed to be a mixture of equal amounts of the (S)-2-cis and (S)-2-trans form of ABA.

### Analysis of anthocyanin levels

Fifty milligram of shoot material were extracted for 1 d at 4°C in 1 ml of 1% (v/v) hydrochloric acid in methanol. The mixture was centrifuged at 18000 × g for 15 min and the absorbance of the supernatant was measured at 530 and 657 nm. Relative anthocyanin concentrations were calculated with the formula [A530 - (0.25 × A657)] to correct for chlorophyll and its degradation products present in the extract.

### Determination of sugars

Plants were harvested at the middle of the light period. Quantification of sugars was conducted using the same shoot material that has been applied to gene expression analysis, quantification of ABA, anthocyanin, and trehalase activity. 50 mg of shoot material were extracted twice with 80% ethanol at 95°C for 30 min, followed by one extraction with 50% ethanol at 95°C for 30 min. The supernatant was used for the determination of glucose, fructose and sucrose by measuring the difference of absorbance at 340 nm in buffer (75 mM HEPES/KOH pH 7, 2.3 mM MgCl, 2.3 mM ATP, 1 mM NADP and glucose-6-phosphate dehydrogenase) after sequentially adding of hexokinase, phosphoglucose isomerase, and invertase. For starch determination, the pellets of the ethanol extraction were solubilized by heating them to 95°C in 0.1 M NaOH for 30 min. After acidification with an HCl/sodium-acetate mixture pH 4.9, part of the suspension was digested overnight with amyloglucosidase and α-amylase. The glucose content of the supernatant was then used to assess the starch content of the sample by measuring the difference in absorbance at 340 nm after adding hexokinase in the same buffer mentioned above.

### Trehalase activity

Aliquots of 70 mg frozen shoot material were resuspended in 1 ml cold extraction buffer (0.1 M MES-KOH pH 6, 1 mM EDTA, 1 mM phenylmethylsulfonyl fluoride, 1% w/v polyvinylphenol, 0.01% Triton X-100, and 1 mM DTT) at 4°C. The suspension was cleared by centrifugation at 13000×g and 4°C for 10 min. Removal of sugars and the subsequent trehalase assay were conducted as described in Delatte et al. (2011).

## Results

### *EXO* alters responses to exogenous sugars

**Figure 1:**
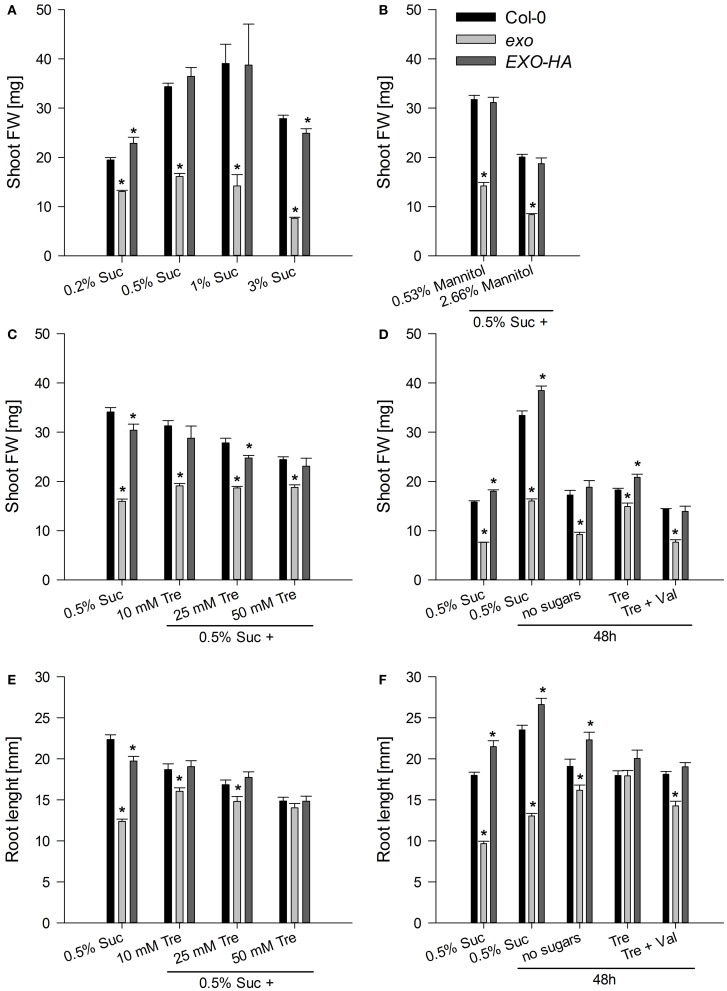
**Fresh weight of shoots and length of roots. (A)** Wild-type, *exo*, and *exo*/35S::EXO-HA plants (termed EXO-HA) were grown on half-concentrated MS medium in the presence of different sucrose concentrations for 14 days. Eight plants were combined for each weight determination. Data are means of 5 values ± SE. Mutant values denoted with an asterisk are significantly different from those of their wild type (*t*-test, *P* < 0.001). Data are representative of three independent experiments with similar results. **(B)** Plants were established in half-concentrated MS medium for 5 days and transferred to medium supplemented with 0.5% w/v sucrose plus 0.53 or 2.66% w/v mannitol to gain osmolarities of the medium that approximate 1 and 3% w/v sucrose, respectively. Data are representative of three independent experiments with similar results. **(C)** Plants were established as described in **(B)** and transferred to half-concentrated MS medium supplemented with 0.5% sucrose plus 0, 10, 25, or 50 mM trehalose for 9 days. Data are representative of three independent experiments with similar results. **(D)** Plants were established in half-concentrated MS medium supplemented with 0.5% sucrose. 12-day-old plants were transferred to medium supplemented with 0.5% sucrose, no sugar, 25 mM trehalose (Tre), or 25 mM Tre and 10 μM validamycin A (Val), a potent trehalase inhibitor, and harvested 2 days later. Data are representative of three independent experiments with similar results. **(E)** Root length of the same plants as described in **(C)**. **(F)** Root length of the same plants as described in **(D)**.

**Figure A1:**
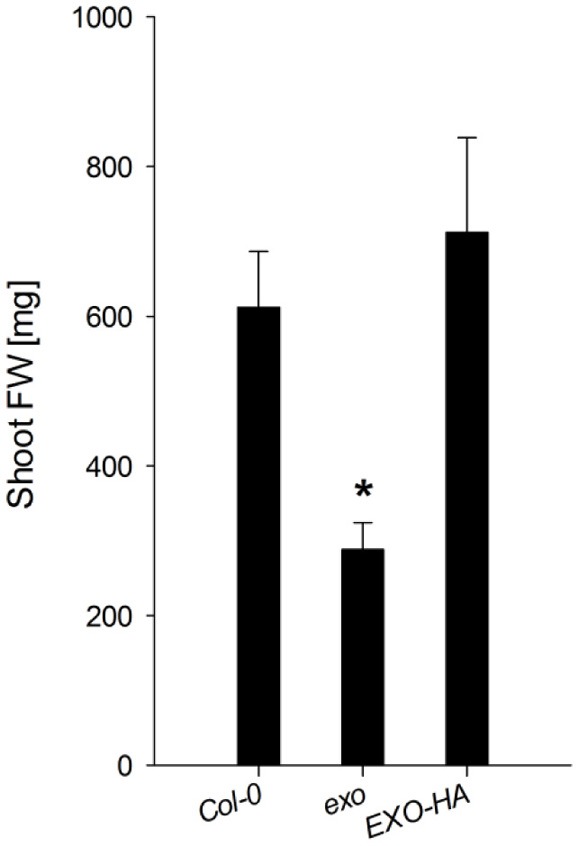
**Shoot fresh weight of wild-type, *exo*, and *exo*/35S::EXO-HA plants.** Plants were grown in soil under long-day conditions and harvested after 35 days. The mutant value denoted with an asterisk is significantly different from the wild-type value (*t*-test, *P* < 0.001).

To test growth in the presence of exogenous sugar, wild-type and *exo* plants were grown in half-concentrated Murashige and Skoog (MS) medium supplemented with different sucrose concentrations. When cultivated on 0.5% w/v sucrose, *exo* produced 47% of the wild-type biomass (Figure 1A). This approximates the growth defect of soil-grown *exo* plants (Figure A1 and Schröder et al., 2009). A lower sugar concentration such as 0.2% w/v sucrose caused reduced growth of the wild type, but barely affected growth of *exo* plants (Figure 1A). A high sugar concentration (3% sucrose) caused a 19 and 53% reduction in shoot biomass growth of wild-type and *exo* plants, respectively, indicating that *exo* is more sensitive to high sugar levels in comparison to the wild type. Overexpression of an HA-tagged EXO protein under control of the 35S promoter in the *exo* mutant largely normalized the sucrose response (Figure 1A).

Addition of sucrose increases the osmolarity of the growth medium and conceivably causes osmotic stress. In order to test osmotic stress sensitivity, plants were grown on medium supplemented with 0.5% sucrose and mannitol. Mannitol is a sugar alcohol that is widely used as osmotic agent in plant research. The *exo* mutant produced about 45% of the wild-type biomass in the presence of 0, 0.53, and 2.66% w/v mannitol. That resembles growth inhibition of *exo* on medium with 0.5% sucrose only. Mannitol similarly impaired growth of wild-type and *exo* plants. Thus, the mutant was not hypersensitive to the increased osmotic concentration in the growth medium.

### Sugar-responsive genes reflect sucrose-hypersensitivity of *exo*

Sugar supply affects the expression of a large set of genes. Expression levels of 24 genes were analyzed in response to exogenous sucrose by means of real-time RT-PCR.

**Figure 2:**
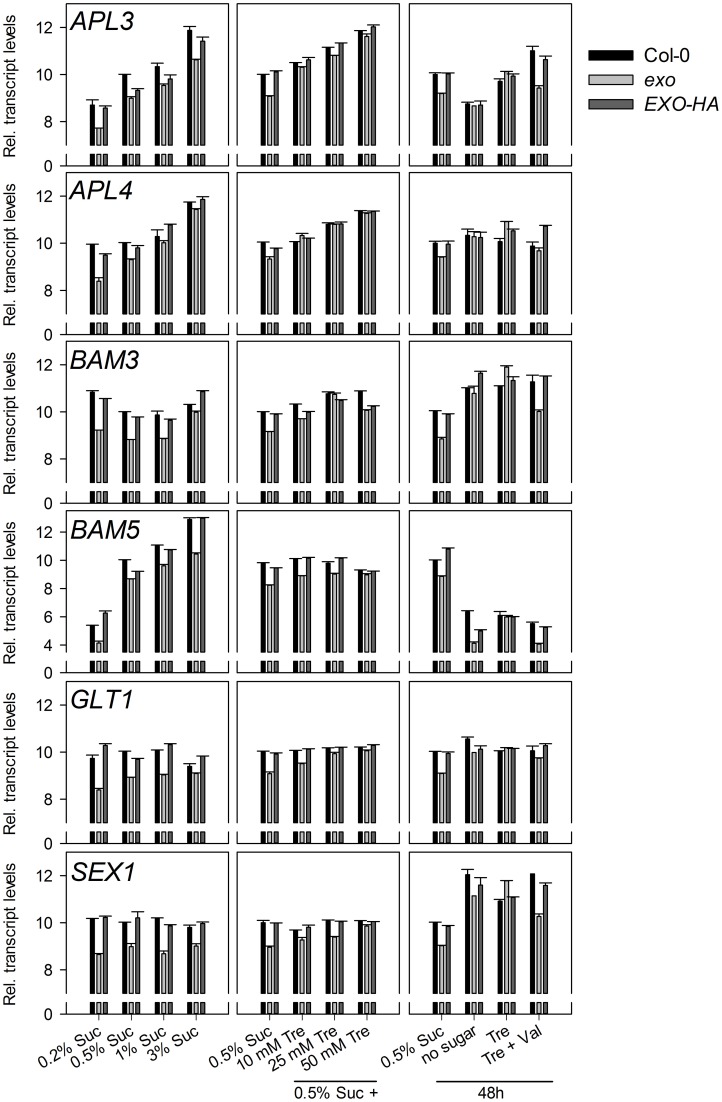
**Expression of starch-related genes.** Quantitative RT-PCR analysis of *APL3*, *APL4*, *BAM3*, *BAM5*, *GLT1*, and *SEX1* transcript levels. Wild-type, *exo*, and *exo*/35S::EXO-HA plants were grown on half-concentrated MS medium supplemented with different sucrose concentrations for 14 days. Alternatively, plants were established in medium supplemented with 0.5% sucrose for 5 days and transferred to medium with 0.5% sucrose and 0, 10, 25, or 50 mM trehalose. For short-term treatments, 12-day-old seedlings were transferred to plates supplemented with 0.5% sucrose, no sugar, 25 mM trehalose (Tre), or 25 mM trehalose plus 10 μmol validamycin A (Tre + Val) and shoot material was harvested 2 days later at the middle of the light period. The cycle threshold values of the *eIF*1α control gene were subtracted from the respective cycle threshold values of the genes of interest. The expression value of the genes at 0.5% sucrose was arbitrarily set to 10, and all other values were adjusted accordingly. Higher numbers indicate higher transcript levels. A difference of one unit indicates a 2-fold change. Error bars indicate SE of the gene of interest in three technical replicates. Data are representative of three independent experiments with similar results.

Six starch-related genes were tested, namely *APL3* and *APL4* (encoding regulatory subunits of ADP-glucose pyrophosphorylase), *BAM3* (also termed *BMY8* or *ctBMY*, encoding a plastidial β-amylase), *BAM5* (also termed *BMY1* or *RAM1*, encoding a cytosolic β-amylase), *SEX1* (also termed *GWD1*, encoding a plastidial α-glucan water dikinase involved in starch degradation), and *GLT1* (encoding a plastidial glucose translocator) (Sebastian and Zeeman, 2012). *APL3*, *APL4*, and *BAM5* expression was induced by exogenous sugar in wild-type and *exo* plants, but expression levels in *exo* were consistently lower. *BAM3*, *SEX1*, and *GLT1* transcript levels were not induced in response to sucrose, but also were lower in *exo* plants (Figure 2). Thus, genes involved in the synthesis and degradation of starch were weaker expressed in *exo*. Anyhow, reduced expression of genes involved in starch biosynthesis such as *APL3* and *APL4* presumably did not limit starch biosynthesis, because starch levels were significantly higher in *exo* in the presence of different sucrose (Figure 4A) and trehalose concentrations (Figures 4B,C). Glucose, fructose, and endogenous sucrose levels were not significantly different from the wild type (data not shown).

**Figure 3:**
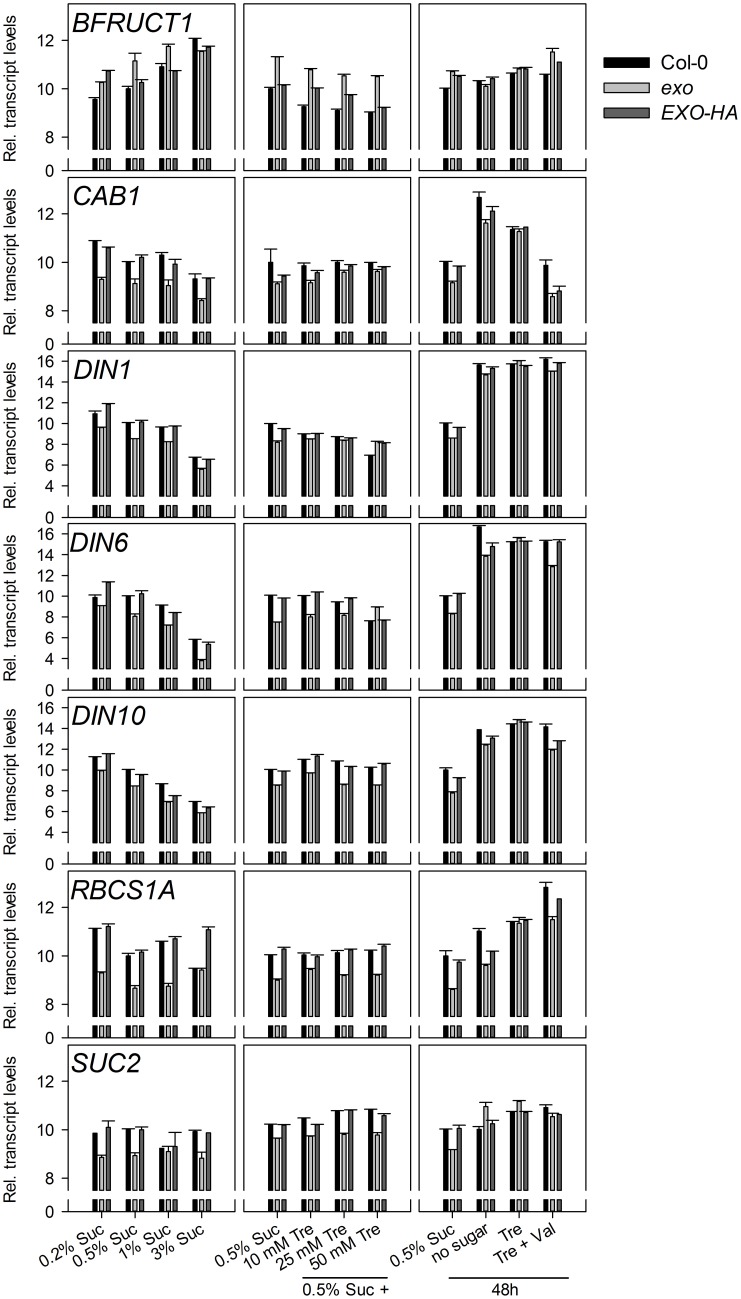
**Expression of sugar-responsive genes.** Quantitative RT-PCR analysis of *BFRUCT1*, *CAB1*, *DIN1*, *DIN6*, *DIN10 RBCS1A*, and *SUC2* transcript levels in wild-type, *exo*, and *exo*/35S::EXO-HA plants shoots. Technical details are as given for Figure 2. Data are representative of three independent experiments with similar results.

The *BFRUCT1*, *CAB1*, *DIN1*, *DIN6*, *DIN10*, *RBCS1A*, and *SUC2* genes have diverging and partly unknown functions in plants, but a common feature is their control by sugars. *CAB1* (also termed *LHCB1.3*, encoding a subunit of light-harvesting complex II), *DIN1* (also termed *SEN1*, senescence associated gene with unknown function), *DIN6* (also termed *ASN1*, encoding an asparagine synthase), *DIN10* (glycosyl hydrolase), and *RBCS1A* (Rubisco small subunit) expression was repressed by sucrose in both genotypes, but transcript levels were consistently lower in *exo* (Figure 3). Expression of the *BFRUCT1* gene (also termed *CWINV1*, encoding a cell wall invertase) was induced by sucrose. In contrast to the other tested genes, *BFRUCT1* transcript levels were slightly elevated in *exo* at 0.2, 0.5, and 1% sucrose (Figure 3). The *SUC2* gene encodes a sucrose transporter that is essential for phloem loading. *SUC2* transcript levels were slightly lower in *exo* in the presence of sucrose. Omission of sucrose normalized *SUC2* expression in the mutant (Figure 3).

Introduction of the 35S::EXO-HA construct into *exo* plants restored wild-type expression levels of both the starch-related and the sugar-responsive genes (Figures 2, 3).

**Figure A2:**
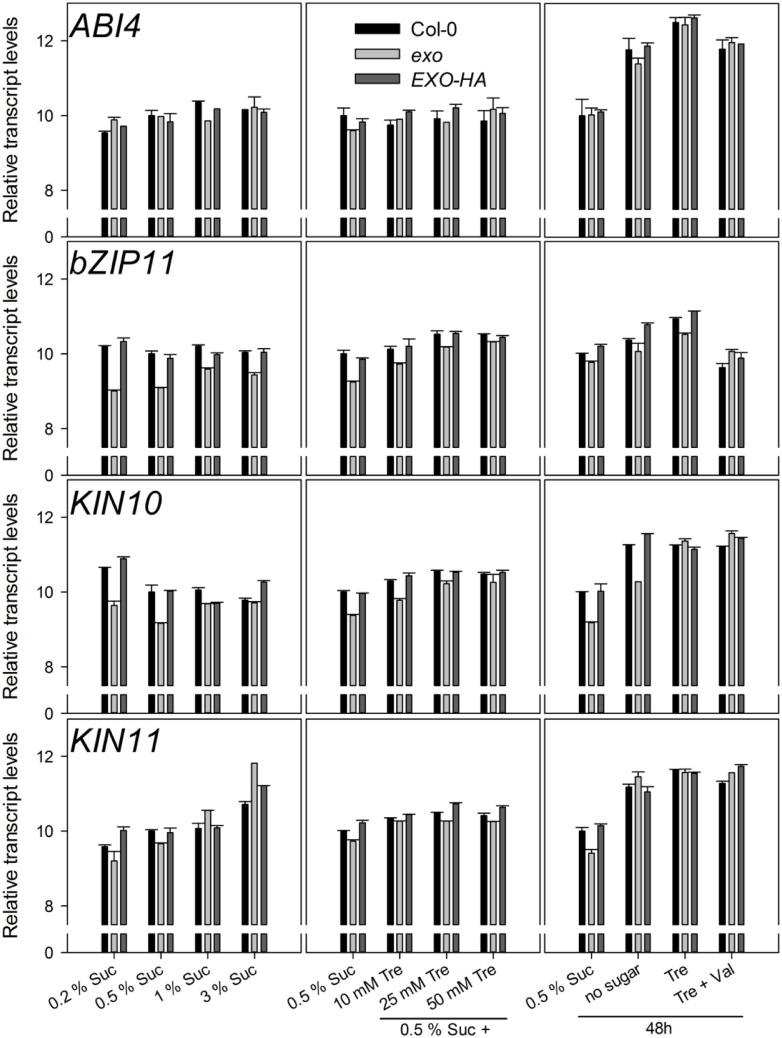
**Relative transcript levels of the *ABI4*, *bZIP11*, *KIN10*, and *KIN11* genes.** Experimental details are described in Figure 2.

**Figure A3:**
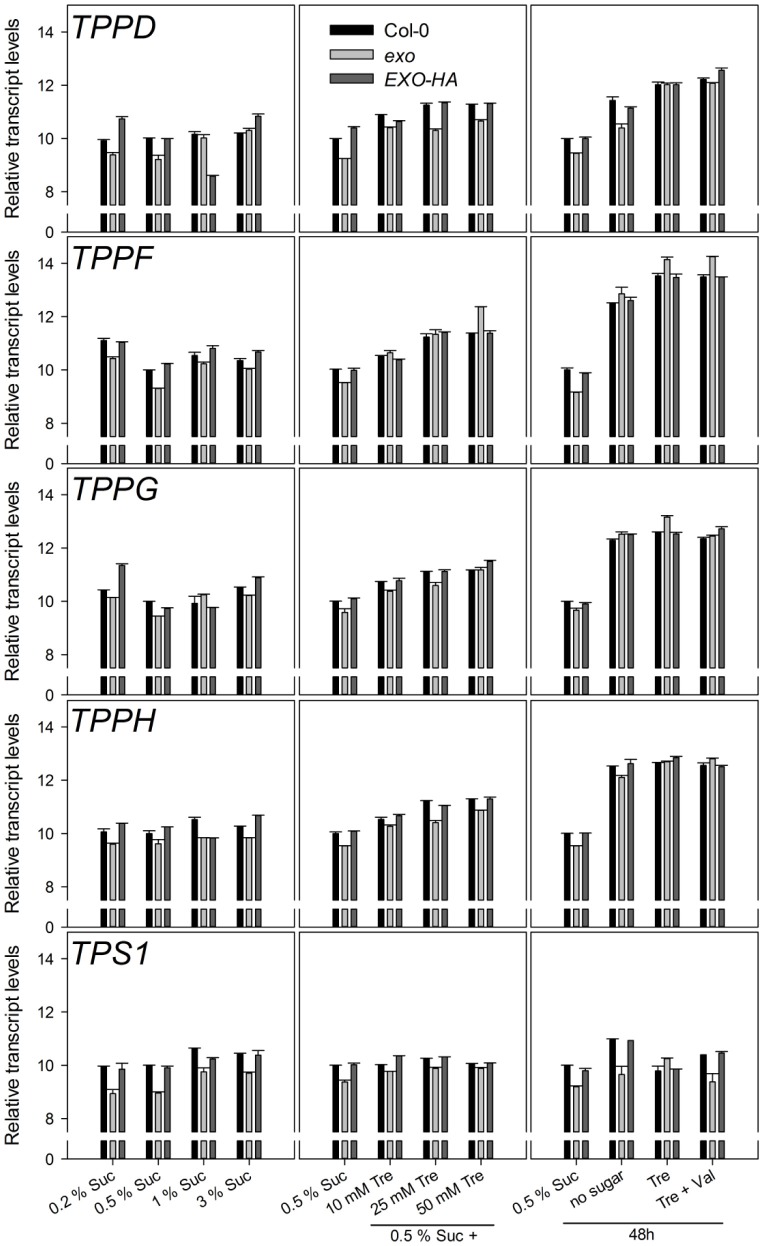
**Relative transcript levels of trehalose-related genes.** Experimental details are described in Figure 2.

**Figure A4:**
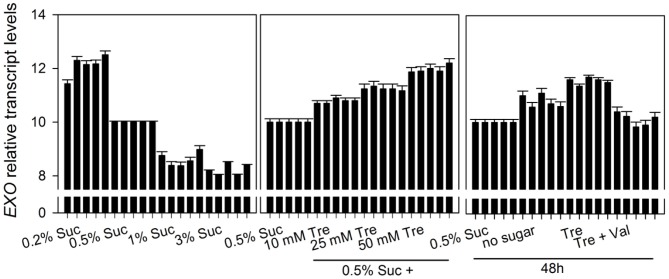
**Relative transcript levels of the *EXO* gene.** Data are derived from an independent biological replica in addition to data given in Figure 5. The 0.5% Suc condition was used as reference condition. Every condition is represented by five columns. The first column of each condition represents cycle threshold values normalized to *Act2*, the second column to *eIF*1α, the third column to *eIF*4α, the fourth column to *GAPC-2*, and the fifth column to *UBQ10*, respectively. Experimental details are described in Figure 2.

**Figure A5:**
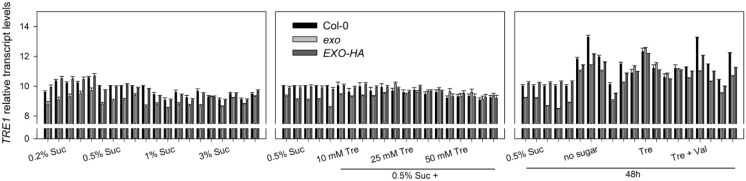
**Relative transcript levels of the *TRE1* gene.** Data are derived from an independent biological replica in addition to data given in Figure 9. Each condition is represented by five sets of three columns (Col-0, *exo*, and EXO-HA). The first set of columns per condition represents cycle threshold values normalized to *Act2*, the second set of columns to *eIF*1α, the third set of columns to *eIF*4α, the fourth set of columns to *GAPC-2*, and the fifth set of columns to *UBQ10*, respectively. Experimental details are described in Figure 2.

The *KIN10* (*SnRK1.1*) and *KIN11* (*SnRK1.2*) kinases and the *bZIP11* and *ABI4* transcription factors play pivotal roles in the control of metabolism. Transcript levels of these genes were tested in wild-type and *exo* plants. Only minor or no differences were observed (Figure A2). We also tested the transcript levels of genes involved in trehalose metabolism. Apart from *TPS1*, transcript levels were similar in wild-type and *exo* plants (Figure A3).

The *EXO* gene itself is controlled by the C status. We previously reported that *EXO* expression is induced in the night, by C starvation, and by a low atmospheric CO_2_ concentration (Schröder et al., 2011). In line with these findings, *EXO* transcript levels were repressed by exogenous sucrose (Figure 5A). Trehalose feeding caused stronger *EXO* expression (Figures 5B,C).

### ABA and anthocyanin levels are elevated in *exo*

Sugars interact with phytohormones to control adaptation to stress and growth. A relationship between ABA and sugar responses was identified by means of mutant analyses. For example, sugar supply causes ABA accumulation in Arabidopsis seedlings (Arenas-Huertero et al., 2000; Huijser et al., 2000; Laby et al., 2000; Cheng et al., 2002). ABA levels were measured in *exo* and wild-type plants grown in medium supplemented with 0.2, 0.5, and 1% sucrose. ABA levels in *exo* were approximately 90% above the wild-type level (Figure 6A). In order to test whether ABA sensitivity was also altered in *exo*, the effects of synthetic ABA on root length and shoot biomass were analyzed. Four-day old seedlings were transferred to medium supplemented with different ABA concentrations as described in Lisso et al. (2011). Shoot biomass of *exo* seedlings in response to exogenous ABA was similarly impaired in comparison to the wild type (data not shown). In contrast to wild-type roots, *exo* roots were barely affected by exogenous ABA (Figure 6B).

Sucrose is an effective inducer of anthocyanin production in Arabidopsis seedlings through the activation of biosynthetic genes (Teng et al., 2005; Das et al., 2012).

Anthocyanin levels were elevated in the *exo* mutant at all tested sucrose levels (Figure 7A). On 0.5% sucrose, *exo* exhibited approximately a twofold anthocyanin concentration in comparison to the wild type. The complemented *exo* mutant had approximately wild-type anthocyanin levels.

### Sugar analogues trigger wild-type growth responses in *exo*

The effects of 2-deoxy-glucose, mannose, and palatinose on biomass production were tested. 2-deoxy-glucose is a glucose analogue that becomes phosphorylated by hexokinases and blocks seedling development (Gibson, 2000). Mannose is an intermediate in sugar metabolism, and also acts as a glucose analogue that is phosphorylated by hexokinases. Feeding mannose inhibits development of Arabidopsis seedlings (Pego et al., 1999). Palatinose is a non-metabolisable structural sucrose isomer (Loreti et al., 2001). The observed relative growth responses to the three compounds were identical in *exo* and the wild type in comparison to the control situation, 0.5% sucrose. No differences in the relative growth inhibition were observed (Figure 8), suggesting that major hexose- and sucrose-dependent signaling pathways are intact in *exo*.

### Exogenous trehalose largely rescues the *exo* mutant

Trehalose is a glucose disaccharide that has physiological effects on growth and carbon allocation in plant seedlings. In addition, trehalose was reported to enhance abiotic stress resistance in plants. However, trehalose presumably does not function as compatible solute and stress protectant in Arabidopsis, because the endogenous concentrations are low (Vogel et al., 2001). Trehalose feeding inhibits growth of Arabidopsis seedlings, causes anthocyanin accumulation, and alters starch levels in several plant organs (Wingler et al., 2000; Aghdasi et al., 2010). The mode of action of growth inhibition includes the accumulation of the signaling compound T6P (Schluepmann et al., 2004).

Plants were established on medium supplemented with 0.5% sucrose. After 5 days, plants were transferred on medium supplemented with 0.5% sucrose and 0 to 50 mM trehalose. In the absence of trehalose, *exo* plants produced 47% of the biomass of the wild type (Figures 1A,C). Addition of trehalose impaired growth of the wild type. For example, addition of 25 mM trehalose in the presence of 0.5% sucrose caused about 20% reduction in biomass production and root length (Figures 1C,E). In contrast, addition of trehalose had positive effects on *exo* plants and partly normalized shoot and root growth (Figures 1C,E).

The inhibitory effect of trehalose is curbed by sugars, because growth of wild-type seedlings on trehalose is restored if sucrose is supplied simultaneously with trehalose (Schluepmann et al., 2004). To investigate the effect of trehalose on growth in the absence of sucrose, and to assess the full impact of trehalose, established seedlings were transferred to different media and allowed to grow for 2 days. In the presence of 0.5% sucrose, all genotypes showed shoot and root growth during the two-day period, demonstrating that the transfer process to new growth medium is not destructive (Figures 1D,F, see outermost left columns). The absence of sucrose resulted in 48% and 42% shoot biomass reduction in wild-type and *exo* plants, respectively (Figure 1D). Root growth of wild-type plants also was reduced in the absence of sucrose. In contrast, *exo* root length was elevated in the absence of sugars (Figure 1F). Supply of trehalose in the absence of sucrose largely normalized *exo* shoot and root growth, but did not positively affect wild-type growth (Figures 1D,F).

**Figure 4:**
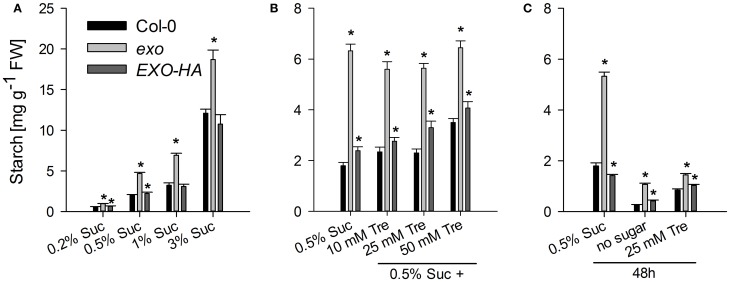
**Starch levels. (A)** Wild-type, *exo*, and *exo*/35S::EXO-HA plants were grown in the presence of different sucrose concentrations. Growth conditions are as given for Figure 1A. Shoot material was harvested at the middle of the light period. Starch levels are given as mean of three determinations ± SE. Mutant values denoted with an asterisk are significantly different from those of their wild type (*t*-test, *P* < 0.05). Data are representative of three independent experiments with similar results. **(B)** Growth conditions are as described in Figure 1C. **(C)** 12-day-old seedlings were transferred to medium supplemented with 0.5% sucrose, no sugar (neither sucrose nor trehalose), or 25 mM trehalose (Tre) and shoots were harvested 2 days later at the middle of the light period.

The rescue of the growth phenotype by exogenous trehalose was also reflected at the molecular level. Most starch-related and sugar-responsive genes were normally expressed in *exo* upon trehalose feeding (Figures 2, 3). Short-term treatments with 25 mM trehalose in the absence of sucrose normalized anthocyanin levels in the mutant (Figure 7C). The altered response of *exo* to trehalose becomes also evident by comparing the starch levels. The wild type accumulated starch in response to trehalose, whereas *exo* starch levels did not increase upon long-term trehalose (Figure 4B) and partly normalized to the wild-type level upon short-term trehalose application (Figure 4C). Thus, growth of the *exo* mutant is promoted rather than impaired by trehalose.

**Figure 5:**
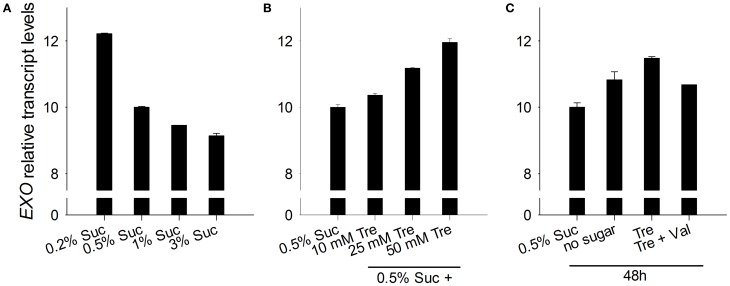
***EXO* expression in response to sucrose and trehalose. (A)** Quantitative RT-PCR analysis of relative *EXO* transcript levels in wild-type shoots. Wild-type seedlings were grown on half-concentrated MS medium as described in Figure 1A. Technical details are as given for Figure 2. Data are representative of three independent experiments with similar results. **(B)** Seedlings were established as described in Figure 1C. **(C)** Seedlings were established as described in Figure 1D.

### Validamycin a prohibits rescue of *exo* by trehalose

Arabidopsis trehalase occurs in multiple tissues and is highly active in flowers and siliques. Low trehalase activity was detected in roots (Müller et al., 2001). The Arabidopsis genome encodes a single trehalase termed *TRE1* that is a plasma membrane-bound enzyme with extracellular activity (Frison et al., 2007). Altering trehalase activity barely changed T6P levels in Arabidopsis, indicating that the altered trehalose content caused the phenotypic differences (Van Houtte et al., 2013).

Trehalase activity in *exo* was slightly reduced in medium supplemented with 0.5% sucrose (Figure 9A). Long-term trehalose supply in the presence of 0.5% sucrose induced trehalose activity in wild-type and *exo* plants. Higher trehalose levels completely normalized trehalase activity levels in *exo* (Figure 9A). Short-term trehalose supply in the absence of sucrose caused a massive induction of trehalase activity, most pronouncedly in *exo* (Figure 9A). Thus, trehalose feeding was associated with the induction of trehalase activity.

Validamycin A (Val) is a strong inhibitor of trehalases in plant tissues (Müller et al., 2001). The simultaneous supply of Val prohibited trehalose-dependent normalization of *exo* shoot growth (Figure 1D), root growth (Figure 1F), and transcript levels (Figures 2, 3). Since the inhibition of trehalase prevents cleavage of trehalose into glucose, the extracellular generation of glucose apparently is essential for the normalization of growth and gene expression patterns in *exo*.

## Discussion

### The apoplastic EXO protein modifies intracellular sugar responses

The *exo* loss-of-function mutant is characterized by reduced leaf and root growth. EXO is essential for cell expansion in leaves because epidermis, palisade and spongy parenchyma cells were smaller in *exo* in comparison to the wild type (Schröder et al., 2009). The highly similar EXO-LIKE1 (EXL1) protein promotes growth under C-limiting conditions (Schröder et al., 2011, 2012). The characteristic feature of the EXO and EXO-LIKE proteins is the PHI1 conserved region (Pfam entry PF04674). Its putative N-terminal targeting sequence directs the protein into the apoplast (Schröder et al., 2009). The remaining primary structure does not show similarity to other known protein domains.

**Figure 6:**
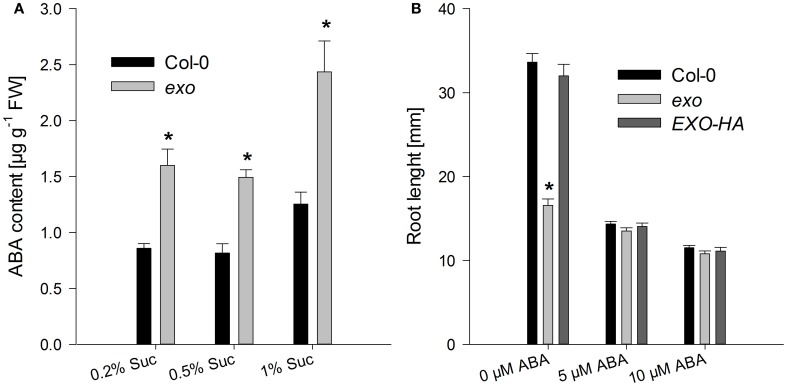
**ABA levels and root growth in response to exogenous ABA. (A)** Wild-type and *exo* plants were grown on half-concentrated MS medium in the presence of different sucrose concentrations for 14 days. Shoot material was analyzed. Results are mean of three determinations ± SE. Mutant values denoted with an asterisk are significantly different from those of their wild type (*t*-test, *P* < 0.001). Data are representative of three independent experiments with similar results. **(B)** Wild-type, *exo*, and *exo*/35S::EXO-HA seeds were allowed to germinate in medium supplemented with 0.5% sucrose. 5-day-old seedlings were transferred to the same medium supplemented with 0, 5, or 10 μM ABA. Root length of 14-day-old plants was determined and is given as mean of 40 roots ± SE. Mutant values denoted with an asterisk are significantly different from those of their wild type (*t*-test, *P* < 0.001). Data are representative of three independent experiments with similar results.

**Figure 7:**
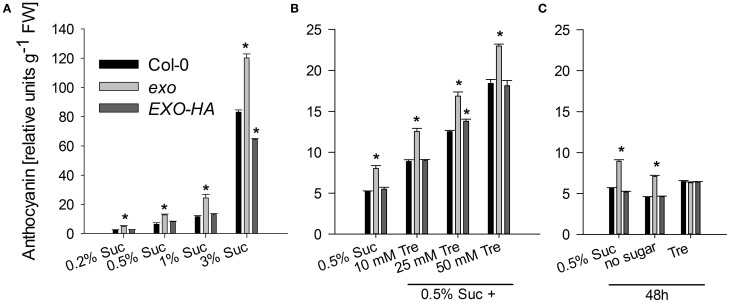
**Anthocyanin levels. (A)** Wild-type, *exo*, and *exo*/35S::EXO-HA plants were grown on half-concentrated MS medium in the presence of different sucrose concentrations as described for Figure 1A. Relative anthocyanin levels were determined in shoot material and are given as mean of 10 quantifications ± SE. Mutant values denoted with an asterisk are significantly different from those of their wild type (*t*-test, *P* < 0.001). Data are representative of three independent experiments with similar results. **(B)** Plants were grown as described for Figure 1C. **(C)** Alternatively, 12-day-old wild-type, *exo*, and *exo*/35S::EXO-HA seedlings were transferred to plates supplemented with 0.5% sucrose, no sugar, or 25 mM trehalose (Tre) and shoots were harvested 2 days later.

The findings shown here suggest that EXO integrates the apoplastic C status such as the level of sugars or sugar-derived signals, intracellular sugar responses, and growth. Loss of *EXO* function caused altered sucrose response of seedling growth (Figure 1), suggesting that the *exo* mutant is impaired in the transport, perception, or signaling of sugars. ABA and anthocyanin levels are associated with the C status (Arenas-Huertero et al., 2000; Cheng et al., 2002; Teng et al., 2005; Das et al., 2012). The growth defect of *exo* is accompanied by elevated ABA (Figure 6), anthocyanin (Figure 7), and starch concentrations (Figure 4). Expression of several sugar-related genes was weaker in *exo* (Figures 2, 3).

**Figure 8:**
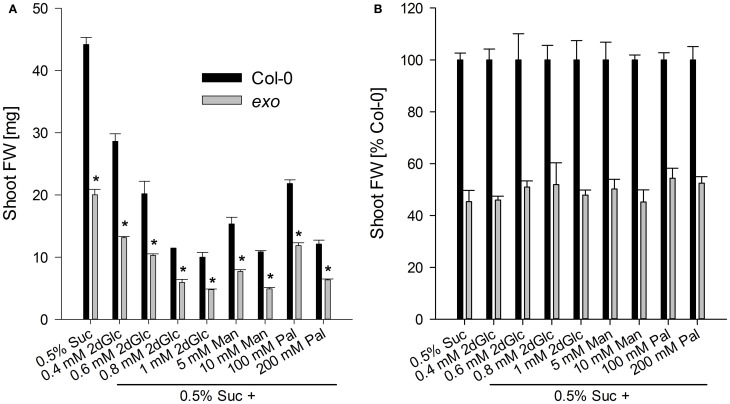
**Shoot biomass in the presence of sugar analogues.** Wild-type and *exo* plants were established in half-concentrated MS medium for 5 days and transferred to medium supplemented with 0.5% sucrose and different concentrations of 2-deoxy-glucose (2dGlc), mannose (Man), or palatinose (Pal). **(A)** Fresh weight of shoots. Seven plants were weighed together and results are mean of five single values ± SE. Mutant values denoted with an asterisk are significantly different from those of their wild type (*t*-test, *P* < 0.001). Data are representative of three independent experiments with similar results. **(B)** Relative fresh weight calculated from data in Figure 8A. Percentage changes were not significantly different in *exo* in comparison to the wild type.

### Exogenous trehalose complements the *exo* mutant

The localization of EXO in the apoplast and wild-type responses to 2-deoxy-glucose and mannose exclude a role as component in hexokinase-dependent signaling pathways. An interaction with plasmamembrane glucose transporter-like sensors or G-protein coupled receptors that represent well-characterized signaling pathways in yeast is speculative, because these systems have been poorly described in plants (Rolland et al., 2006).

**Figure 9:**
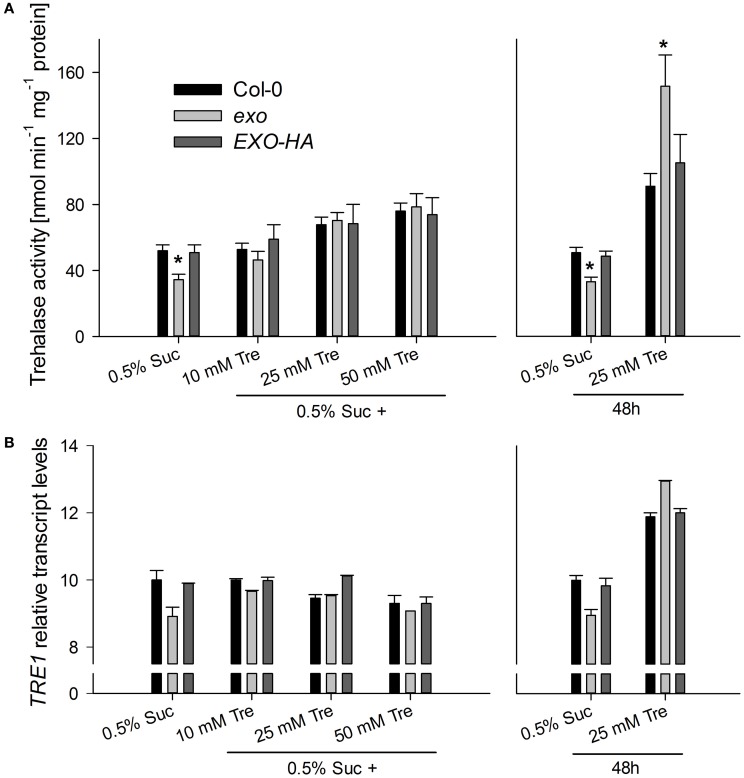
**Trehalase activity and *TRE1* expression. (A)** Wild-type, *exo*, and *exo*/35S::EXO-HA plants were established in half-concentrated MS medium supplemented with 0.5% sucrose for 5 days and transferred to medium supplemented with 0.5% sucrose plus 0, 10, 25, or 50 mM trehalose for 9 days. Alternatively, plants were established in half-concentrated MS medium supplemented with 0.5% sucrose. 12-day-old plants were transferred to medium supplemented with 0.5% sucrose with or without 25 mM trehalose (Tre). Trehalase activity in shoot material is given as mean of three determinations ± SE. Mutant values denoted with an asterisk are significantly different from those of their wild type (*t*-test, *P* < 0.001). Data are representative of three independent experiments with similar results. **(B)** Relative *TRE1* transcript levels in shoots were given as described for Figure 2. Data are representative of three independent experiments with similar results.

Interestingly, the phenotypic changes of *exo* were largely normalized by exogenous trehalose. The disaccharide slightly stimulated growth (Figures 1C–F), normalized sugar-related gene expression patterns (Figures 2, 3), and reduced anthocyanin levels (Figure 7). Thus, either trehalose itself or trehalose-derived compounds complement the *exo* mutant. Two lines of arguments suggest that extracellular trehalose-hydrolysis is essential for the complementation. First, complementation of the mutant by trehalose feeding was associated with enhanced trehalase activity (Figure 9). Second, simultaneous feeding of the trehalase inhibitor validamycin A reversed the positive trehalose effects (Figures 1–3).

Exogenous trehalose usually inhibits Arabidopsis seedling growth. Intracellular T6P accumulation was identified as a major cause for this (Schluepmann et al., 2004). Plants with enhanced trehalase activity grow better on trehalose. This was demonstrated for plants expressing an *E. coli* trehalase in the cytoplasm (35S::treF) and for plants expressing the Arabidopsis TRE1 enzyme (35S::TRE1) (Schluepmann et al., 2004; Aghdasi et al., 2010; Delatte et al., 2011; Van Houtte et al., 2013). 35S::treF plants hydrolyze trehalose that has entered the cell, whereas 35S::TRE1 seedlings could metabolize trehalose before it passes the plasma membrane. Although 35S::treF plants displayed higher trehalase activity than 35S::TRE1 plants, they exhibited reduced growth compared to 35S::TRE1 seedlings (Van Houtte et al., 2013). Apparently, it is advantageous when the exogenous trehalose does not enter the cytoplasm, because the intracellular C-metabolism is not impaired by trehalose and trehalose-derived signals such as T6P.

The generation of glucose from trehalose presumably complements the *exo* mutant. The mutant could benefit from the generation of glucose for metabolic purposes. Alternatively, enhanced trehalase activity may trigger extracellular signaling processes.

Recent data revealed that trehalose metabolism rather than the trehalose itself is essential for proper stomatal function and survival of drought stress (Van Houtte et al., 2013). This finding was somewhat unanticipated, because trehalose was widely seen as a compatible solute and stress protectant (Vogel et al., 2001; Van Houtte et al., 2013). However, enhanced trehalase activity *per se* is not beneficial under all conditions. *TRE1* overexpressors exhibited reduced growth in non-stress conditions (Van Houtte et al., 2013). Restricted growth of the *exo* mutant on sucrose did not result from elevated trehalase activity because *exo* displayed reduced trehalase activity when grown on sucrose (Figure 9).

## Conclusions

The phenotype of the *exo* mutants points to the importance of the extracellular C status. Although the apoplastic C-metabolism and C-signaling processes are largely unknown, the altered sucrose and trehalose responses in *exo* suggest a role of the EXO protein in the control of the extracellular C-metabolism or C-signaling. Loss of *EXO* function causes reduced growth, impaired expression of starch-related and sugar-responsive genes, accumulation of ABA, anthocyanin, and starch. The phenotypic changes can partly be normalized by trehalose feeding. The poor understanding of extracellular processes complicate the understanding of EXO action. The availability of more data about these processes will allow building new hypothesis and moving toward targeted approaches.

### Conflict of interest statement

The authors declare that the research was conducted in the absence of any commercial or financial relationships that could be construed as a potential conflict of interest.

## Appendix

**Table A1:** **Primer sequences used for quantitative RT-PCR**.

**Gene**	**AGI code**	**Forward primer (5′–3′)**	**Reverse primer (5′–3′)**
*ABI4*	At2g40220	CGG TGG GTT CGA GTC TAT CAA	ACC CAT AGA ACA TAC CGG ATC AA
*APL3*	At4g39210	AAA CCG AGA AGT GCC GGA TTG	GTT GGA TGC TGC ATT CTC CCA AG
*APL4*	At2g21590	CGT GTG CAC CAG TTA GTG AAA	AAA TGG ATT ACA CGC CCA CCT C
*BAM3*	At4g17090	AAA GCA CGG TCT CAA ACT CCA G	GGG ATA CTG CAA GAG TCT CCT ACG
*BAM5*	At4g15210	ATA CCG ACA ATA CAG CTG AAG CC	TCC ATG CCT TGC TCA GTA CCT C
*BFRUCT1*	At3g13790	ATT CAG TGG CCG GTT AGG GAA G	CAA TAC TTC TAC ATC CGC CTG TGC
*bZIP11*	At4g34590	ACA ACA TGG GCA TGT GTT CG	GCC ATG AGA GGC TGG TTC AT
*CAB1*	At1g29930	TGC ACT ACT CAA CCT CAA TGG C	AAA GCT TGA CGG CCT TAC CG
*DIN1*	At4g35770	TCT TCT CAC TGC TGG CTT CAC G	TCT CTG TCC AAG CGA CGT ATC C
*DIN6*	At3g47340	AAG GTG CGG ACG AGA TCT TTG G	ACT TGT GAA GAG CCT TGA TCT TGC
*DIN10*	At5g20250	CTT CTT CGC TTT CTG GCA TTG TTC	GCT GCT TCG CTT CTC TTG TAT GAC
*EXO*	At4g08950	TGG TAC GGC AAA TTC AAA CC	GTG GCA ACA GAT GGT TGG TG
*GLT1*	At5g16150	AGC AGC CGC TAC TGG AAA GTT G	TCT GAT TGG ATT CCC GCA CTA CG
*KIN10*	At3g01090	CAA CCG AAC CCA GAA TGA TGG C	ATA ACC ACT AGA GGC ACG GAA ACG
*KIN11*	At3g29160	CCG TGT TCC AAG TGG CTA TC	ACA TGT GCA GGA ATC CAG TG
*RBCS1A*	At1g67090	ACC TTC CTG ACC TTA CCG ATT CCG	TCA CGG TAC ACA AAT CCG TGT CG
*SEX1*	At1g10760	TGT TGG GCT ACC CAA GCA AAC C	TCC TCG TCC ATT GGT ACA CTG TCG
*SUC2*	At1g22710	CTA GCC ATT GTC GTC CCT CAG ATG	ACC ACC GAA TAG TTC GTC GAA TGG
*TPPD*	At1g35910	TGG AAG ATG CCG TGA TAA GGT TCG	TGA TGT CCA TTC CAT GGC TAC CTG
*TPPF*	At4g12430	TTC TCC ACC GCG TAA GAA GC	CGC CGA AGG ATA TTT CAC CA
*TPPG*	At4g22590	ATG CGT GCT GCT GTG AAA GA	TGT CCA TCC CGT GAC TAC CC
*TPPH*	At4g39770	GAT GAT CGC ACC GAC GAA GAT G	AAC CTC ATC GGG TTC TTG CAG TG
*TPS1*	At1g78580	TGG AAT GGG TTG ATA GCG TAA AGC	CGC GAG TTT CAA AGT GTG ACC TG
*TRE1*	At4g24040	AGC GAG AGA GAA AGC GTT TC	CCT TCC ATG TCT CAG ATT C
*Act2*	At3g18780	GTT GAC TAC GAG CAG GAG ATG GAA A	CAT TTT CTG TGA ACG ATT CCT GGA C
*eIF1*α	At5g60390	TTG ACA GGC GTT CTG GTA AGG	CAG CGT CAC CAT TCT TCA AAA A
*eIF4*α	At3g13920	CAC GTC AGT TTG ACC AGA AAC TCA A	ATT GTT GCG GAG AAC ACA CCA ACT T
*GAPC-2*	At1g13440	TTG GTG ACA ACA GGT CAA GCA	AAA CTT GTC GCT CAA TGC AAT C
*UBQ10*	At4g05320	ACA CTT CAC TTG GTC TTG CGT	AGT CTT TCC GGT GAG AGT CTT CA
